# RETRACTED: Azizi et al. Cellulose Nanocrystals/ZnO as a Bifunctional Reinforcing Nanocomposite for Poly(vinyl alcohol)/Chitosan Blend Films: Fabrication, Characterization and Properties. *Int. J. Mol. Sci.* 2014, *15*, 11040–11053

**DOI:** 10.3390/ijms26062525

**Published:** 2025-03-12

**Authors:** Susan Azizi, Mansor B. Ahmad, Nor Azowa Ibrahim, Mohd Zobir Hussein, Farideh Namvar

**Affiliations:** 1 Department of Chemistry, Faculty of Science, Universiti Putra Malaysia, 43400 UPM Serdang, Selangor, Malaysia; norazowa@science.upm.edu.my; 2 Materials Synthesis and Characterization Laboratory, Institute of Advanced Technology, Universiti Putra Malaysia, 43400 UPM Serdang, Selangor, Malaysia; mzobir@science.upm.edu.my; 3 Institute of Tropical Forestry and Forest Products, Universiti Putra Malaysia, 43400 UPM Serdang, Selangor, Malaysia; farideh.namvar@gmail.com; 4 Mashhad Branch, Islamic Azad University, Mashhad 9187147578, Iran

The journal retracts the article, “Cellulose Nanocrystals/ZnO as a Bifunctional Reinforcing Nanocomposite for Poly(vinyl alcohol)/Chitosan Blend Films: Fabrication, Characterization and Properties” [1], cited above.

Following publication, concerns were brought to the attention of the publisher regarding the reliability of the figures presented in this article [1].

Adhering to our complaints procedure, an investigation was conducted by the Editorial Office and the Editorial Board. While the authors cooperated with the Editorial Office during the investigation, they were unable to satisfactorily explain the irregularities present in Figures 3 and 7 or provide raw material suitable for Editorial Board evaluation. As a result, the Editorial Board has lost confidence in the reliability of the findings and decided to retract this publication [1], as per MDPI’s retraction policy (https://www.mdpi.com/ethics#_bookmark30).

This retraction was approved by the Editor-in-Chief of the *International Journal of Molecular Sciences* journal. The authors agreed to this retraction.
